# Transcriptomic changes in relation to early-life events in the gilthead sea bream (*Sparus aurata*)

**DOI:** 10.1186/s12864-016-2874-0

**Published:** 2016-07-26

**Authors:** E. Sarropoulou, A. Tsalafouta, A. Y. M. Sundaram, G. D. Gilfillan, G. Kotoulas, N. Papandroulakis, M. Pavlidis

**Affiliations:** 1 Institute of Marine Biology, Biotechnology and Aquaculture, Hellenic Centre for Marine Research, Gournes Pediados, Heraklion, Crete 71003 Greece; 2 Department of Biology, University of Crete, Heraklion, Crete 70013 Greece; 3 Department of Medical Genetics, Oslo University Hospital and University of Oslo, Oslo, Norway

**Keywords:** Early-life events, Stress, RNAseq, DEG, *Sparus aurata*

## Abstract

**Background:**

Teleosts are exposed to a broad range of external stimuli, which may be either of acute or chronic nature. The larval phase of certain fish species offer a unique opportunity to study the interactions between genes and environmental factors during early life. The present study investigates the effects of early-life events, applied at different time points of early ontogeny (first feeding, flexion and development of all fins; *Phase 1*) as well as on the subsequent juvenile stage after the application of an additional acute stressor (*Phase 2*) in the gilthead sea bream (*Sparus aurata*), a commercially important European aquaculture species. Animal performance, the cortisol response and gene expression patterns during early development as well as on the subsequent phases (juveniles) after the application of additional acute stressors were investigated.

**Results:**

Significant differences on fish performance were found only for juveniles exposed to early-life events at the phase of the formation of all fins. On the transcriptome level distinct expression patterns were obtained for larvae as well as for juveniles with the most divergent expression pattern found to be again at the phase of the development of all fins, which showed to have also an impact later on in the acute stress response of juveniles.

**Conclusions:**

The present study showed that applying an early-life protocol, characterized by the unpredictable, variable and moderate intensity of the applied stimuli provides a relative realistic model to evaluate the impact of daily aquaculture practices on fish performance. In addition, the power of investigating global gene expression patterns is shown, providing significant insights regarding the response of early-life events during development and as juveniles after the application of extra acute stressors.

**Electronic supplementary material:**

The online version of this article (doi:10.1186/s12864-016-2874-0) contains supplementary material, which is available to authorized users.

## Background

Early life history plays an important role on development, coping ability, health and performance of an individual. Teleosts are exposed throughout their life to a broad range of external factors which may be climatic, e.g. extreme cold or heat, nutritional as well as of social nature [1–3]. Cultured teleost species are exposed to an additional range of noxius stimuli and / or stressors such as overcrowding, confinement, chasing, netting, unsuitable water temperatures, poor water quality and overfeeding. The response to environmental parameters is considered a basic element of animal adaptation to various challenges and can affect all forms of animal performance. It further involves complex interactions, from the molecular to the systems level, and may render teleosts more susceptible to infections, inhibit growth or possibly result in reproductive failure [4].

It has also been shown that the life history of an animal plays an important role in stress response (for a review see [5]). Yet, differences are observed according to the nature, intensity, duration and predictability of the applied stressors as well as between individuals and between different species [6–8]. Besides the variety of parameters concerning external stimuli, a broad range of different responses to external stimuli have also been described, which may be of an adaptive or maladaptive nature [6, 7, 9]. The latter influences survival, reproductive capabilities, general fitness and immune response [6, 7, 10, 11]. It has also been shown, that in order to ensure decent animal welfare a certain allostatic load is required [12]. Nevertheless, up until now, only scarce information has been available dealing how established ordinary rearing methods during early life stages impacts the brain function, learning ability and coping styles of fish and more importantly, if they are of adaptive or maladaptive nature.

Studies in teleosts showing differential gene expression after stress exposure have been performed on core genes such as those involved in the classical regulation of the hypothalamic-pituitary-interrenal axis (e.g. [5, 7, 13, 14]). These core genes, assumed to be involved in stress response, have been shown to be highly conserved across teleosts [15, 16]. Further studies in teleosts applying high throughput expression analysis methods however, have identified several genes not previously known to be responsive to external stimuli. These studies broadly classified the majority of differentially expressed transcripts as genes playing an important role in metabolism, immunity and reproduction [17–19].

Early life patterns of fish species offer a unique opportunity to study the interactions between genes and environmental factors. In addition, for fish under captivity, larval stages represent one of the most critical periods to ensure high performance and superior quality in the subsequent developmental stages of the life cycle [20]. The first attempts to investigate stress response in gilthead sea bream (*Sparus aurata*) juveniles using microarray technology for high throughput expression analysis, identified transcripts mainly involved in gluconeogenesis, glycolysis and in the respiratory chain [21]. Further expression analysis in liver of the gilthead sea bream after confinement stress, as well as after low temperature exposure, showed that genes involved in metabolic pathways as well as the endoplasmic reticulum are of significance regarding the transcriptional regulation of stress response [22, 23].

However, the effect of early life events on fish performance has not yet been thoroughly examined. Here we investigate for the first time the transcriptome, the cortisol stress response and the performance of gilthead sea bream larvae exposed to unpredictable environmental, husbandry and social events of mild intensity at different time points of early ontogeny (first feeding, flexion and development of all fins – hereafter referred to as *Phase 1*) as well as on the subsequent juvenile stage after the application of an additional acute stressor (referred to as *Phase 2*) (Additional file 1: Figure S1).

## Methods

All procedures such as handling and treatment of fish used during this study were approved by the HCMR institutional animal care and use committee following the three Rs (Replacement, Reduction, Refinement) guiding principles for more ethical use of animals in testing, first described by Russell and Burch in 1959 (EU Directive 2010/63). All experiments/methods in the present study were performed in accordance with the approved guidelines and regulations.

### Larval rearing

Larval rearing was performed in 500-L cylindro-conical tanks. During the first phase feeding was based on daily supplementation of zooplankton (enriched rotifers and *Artemia nauplii*) and also phytoplankton for a period of 2 weeks. During the second phase feeding was based on *Artemia nauplii* and the weaning to artificial diets was completed. Tanks were coupled to a biological filter and were initially filled with filtered seawater from a deep well. Water, during embryogenesis, egg hatching and at the autotrophic larval stage, was re-circulated from the bottom of the tank through the biological filter at a rate of 10 % h^−1^ and was progressively increased to 70 % h^−1^. Following first feeding the water renewal in the tanks was set to 20 % h-1 and was gradually increased to 170 % at the end of the experimental period. Aeration was provided by means of a wooden diffuser located in the tank center at a rate of 150–200 ml min^−1^.

### Experimental design

#### Early life events - larvae (Phase 1, P1)

Gilthead sea bream individuals were exposed at different stages of early ontogeny (Additional file **1**: Figure S1) to various events, representing possible stimuli experienced during intensive larvae rearing. All experiments were conducted in duplicate tanks per experimental group. The protocol was based on a previous unpredictable chronic low intensity stress (UCLIS) protocol, developed for the European sea bass (*Dicentrarchus labrax*) [24] and consisted of optical (increase in light intensity from 60 to 200 lux for 15 min; lights on for 0.5 h during night; lights off for 0.5 h during day; exposure to blue or red spectrum for 0.5 h), mechanical (high aeration for 90 sec) and social (presence of novel object for 0.5 h) mild stimuli. Two different types of stimuli were applied randomly on a daily basis for the total period of 14 days, thus fish were kept under a mild unpredictable chronic stress that minimized the potential for habituation. Full spectrum lights (Phillips, TLD 36 W) were used to approximate natural light and transparent filters to produce the blue (maximum absorption spectrum 450–475 nm) and red (maximum absorption spectrum 620–750 nm) spectra. The novel objects used were large sized Lego bricks of intense red and green color. Water samples (1 l) were taken at regular intervals (0, 1, 3, 5, 7, 10 and 14 days) from the rearing tanks of the control and experimental groups. Water samples were used to determine water-born cortisol as a non-invasive method to evaluate stress [24].

During larval rearing and pre-weaning a sample of 10 larvae was taken daily to determine morphological characteristics and record total length, while 2 times per week weight measurements were also performed with a sample of 10 individuals. At the end of larval rearing (60 dph) the biological performance of each group was evaluated by estimating the survival rate and the total growth rate.

For transcriptome analysis three biological replicates of each sampling point including control fish were collected resulting into a total of 18 samples.

#### Exposure to acute stress at juvenile stage (Phase 2, P2)

Post larvae were transferred into 1.5 m^3^ cylindrical tanks for pre-growing. The water used was from a deep well (36 psu, 19 ± 1 °C) and the renewal rate at ~50 % per hour. The photoperiod was set at 12 L : 12D. Individuals were fed with artificial diets (INVE aquaculture S.A) appropriate to their age and size.

At the end of the experimental period of *Phase 2* (i.e. about two months after the end of the early-life events), 40 fish per group were sampled to measure their total length and body weight. In addition a representative sample per group was analyzed to estimate their qualitative characteristics in terms of potential deformities. To evaluate stress two types of control fish were used [24]; one with minimum handling (fish captured with a net immediately, without decrease of the tank’s water level, after the distribution of a small amount of food in the tank), and another with common handling practice (decrease of the water level, crowding and netting). Juveniles were then exposed to an acute stress protocol, consisted of crowding (10 min), chasing (5 min) and air exposure (1 min) (Additional file 1: Figure S1) to evaluate the cortisol stress response between the groups with different early life stress history. Fish were transferred to 70 l buckets where blood was collected at 1 h post-stress. The time-point was chosen based on previous data in gilthead sea bream showing maximum plasma cortisol concentrations at 1 h post-stress exposure [8]. In all groups 10 fish were sampled, euthanized with anaesthetic overdose and whole body samples were collected.

For transcriptome analysis three biological replicates of whole brain tissues were collected from individuals after common handling (control group) and after the acute stress application for each group (“P2-FF”, “P2-FLX”,“P2-FINS”) as well as for juveniles not having experienced early-life events (“Acute”) resulting in a total of 24 samples.

#### Whole body cortisol

Cortisol extraction was performed according to de Jesus et al.[25] and Pavlidis et al. [26]. Briefly, whole-trunk samples were partially thawed on ice and homogenized in 5× (w/v), ice-cold, phosphate-buffered saline (pH 7.4) with a rotor homogenizer. Cortisol was extracted by adding 3 mL of diethyl ether to 2 × 250 μl of homogenate. The liquid phase of the extract was allowed to freeze by placement of the tubes in – 80 °C and the combined diethyl ether layer was transferred into a new tube. The tubes were placed in a 45 °C water bath for 1 h and at room temperature for an additional 3 h in order to allow the ether to evaporate completely. Samples were then reconstituted in 250 μl of an enzyme immunoassay buffer. Cortisol was measured using commercial enzyme immunoassay (EIA) kits (Cayman Chemical, MI, USA).

#### Water-born cortisol release rate

Water samples (1 l) were peristaltically pumped at circa 10 ml min^−1^ through a pre-filter (0.45 *μ*m poresize: AcroCap™, GelmanSciences, Ann Arbor, MI, USA) and then through an activated solid phase extraction cartridge (Sep-pak® Plus C18, Waters, UK). Cartridges were then stored frozen until assayed. Free corticosteroids were subsequently eluted with 4 ml ethyl acetate. Ethyl acetate was evaporated at 45 °C under nitrogen gas and the residue was re-dissolved in 1 ml of EIA buffer. Free cortisol concentrations were measured using commercial enzyme immunoassay (EIA) kits (Cayman Chemical, MI, USA). The amount of hormone (*H*) in ng released over a given time interval (*t*) in h was calculated according to Ellis et al. (2004) [27], by adapting the equation of Adams and Breck (1990) [28], *H*_*t*_ = *V kt(C*_*t*_ − *C*_0_e^−*kt*^*) (*1 − e^−*kt*^*)*^−1^, where *V* is the water volume (i.e., tank volume minus fish biomass), *C*_0_ and *C*_*t*_ are the hormone concentrations at the beginning and end of the sampling period (over a time interval *t*) and *k* is the instantaneous rate of decrease due to dilution from the inflow water. Values for *k* were derived as *R/V*, where *R* is the water inflow rate. The hormone release rate (ng g^−1^ h^−1^) was then calculated from *H*_*t*_ and fish biomass. The hormone release rate (ng g^−1^ h^−1^) was subsequently calculated from the differences in the amount of cortisol between sampling points, fish biomass and time, as described in Fanouraki et al. [29] and in larval rearing of European sea bass [24].

#### Statistical analysis

All statistical analyses were performed with SigmaPlot 11.0 (Jandel Scientific). Data are presented as mean ± standard deviation (SD). For comparison of the growth rates between the different conditions during the larval phase, multiple regression analysis was used. This method was applied for the comparison of both the total length (TL) and the wet weight (WW), for which the latter dataset was ln-transformed. Statistical comparisons of (i) total length and body weight at two months after the end of the experimental trial, were made using one-way ANOVA. Statistical comparisons of (ii) temporal patterns of water cortisol release rates between the different groups within each respective developmental phase, for which the stress protocol was applied, but also among the different developmental phases and (iii) whole body cortisol levels between minimum handling, common handling and acute stressed fish among the different groups were made using two-way ANOVA. Tukey’s post-hoc tests was used to assess the level of significance. The significance level cut off was *p* < 0.05.

### Transcriptome sequencing and differential expression analysis

#### Messenger RNA extraction

Messenger RNA was extracted from all samples using the Nucleospin miRNA Kit (Macherey-Nagel GmbH & Co. KG, Duren, Germany) according to manufacturer’s instructions. In brief, larvae and brain tissues were disrupted in liquid nitrogen using mortar and pestle, dissolved in lysis buffer and subsequently passed through a 23-gauge (0.64 mm) needle 5 times to homogenize the mixture. RNA quantity was determined with a NanoDrop ND-1000 spectrophotometer (NanoDrop Technologies Inc, Wilmington, USA) and the quality was further evaluated by agarose (1 %) gel electrophoresis and Bioanalyzer 2100 (Agilent, USA) using RNA Nano Bioanalyzer chips.

#### RNA sequencing

Sequencing libraries were prepared from mRNA using TruSeq v2 RNA reagents (Illumina, USA), with 4 min fragmentation and 15 cycles PCR. Indexed libraries were sequenced over a total of five lanes on a HiSeq 2000 (Illumina, USA), employing 125 bp paired end reads.

#### Quality control and de novo assembly

Quality control on the raw data was performed for all reads using FastQC (version 0.10.0; http://www.bioinformatics.babraham.ac.uk/projects/fastqc). Low quality reads were discarded using default parameters in Trimmomatic software v0.33 [30]. Transcriptome assembly was performed using Trinity v 2012-06-08 [31] with reads resulting from paired end libraries.

#### Differential expression analysis

Paired end reads of each developmental stage were mapped to the reconstructed transcriptome using Bowtie2 v2.2.3 and BBMap v34 [32, 33], allowing a maximum of 3 mismatches per read. For quantification of read abundances RSEM (v1.2.3) [34] was applied and transcripts represented less than once per million mappable reads were excluded from the following analysis. Differential expression was assessed using R Bioconductor package EdgeR, v 3.8.5 [35]. Three sets of potentially differential expressed transcripts were chosen for downstream analysis with the first set having a threshold of *p*-value < 0.001, the second FDR value < 0.05 and the third set representing the most stringent one where in either case all three replicates are not expressed at all (no transcripts identified).

#### Cluster analysis

Hierarchical cluster analysis of significantly differentially expressed transcripts was performed using the function heatmap.2 of gplots in R (v 3.0.2) and the optimal number of clusters were obtained by generating a scree diagram plotting the distance against the cluster numbers. K-means clustering method using 100 iteration in SPSS statistical package (v 12.0) was further applied to partition transcripts according to their expression pattern. The number of centers was determined by the plot of the within groups sum of squares by number of clusters. Corroboration of the cluster assignments was assessed using canonical discriminant analysis. Principal component analysis (PCA) with maximal iteration 25 was computed with the SPSS statistical package (v 12.0) as well as with the prcomp function using the default parameters in R (v 3.0.2). In brief, Eigenvalues greater than 1 were extracted, thus the first three principal components form the extracted solution were kept for further analysis accounting for almost 50 % of the variability. To assure suitability of PCA analysis, Kaiser-Meyer-Olkin measurements as well as Barletts Test of Sphericity were performed. The first three components were visualized by a 3D plot in R (v 3.0.2).

#### Blast, annotation and classification

Transcripts significantly expressed in any of the sampling points were annotated using BLAST search (version 2.2.25) [36] against the non-redundant protein database and non-redundant nucleotide database. Results were further analyzed with Blast2GO software [37] in order to determine the GO terms: cellular component, molecular function and biological process.

## Results

### Effect of treatment on water-born cortisol concentrations and fish performance

**Fig. 1:**
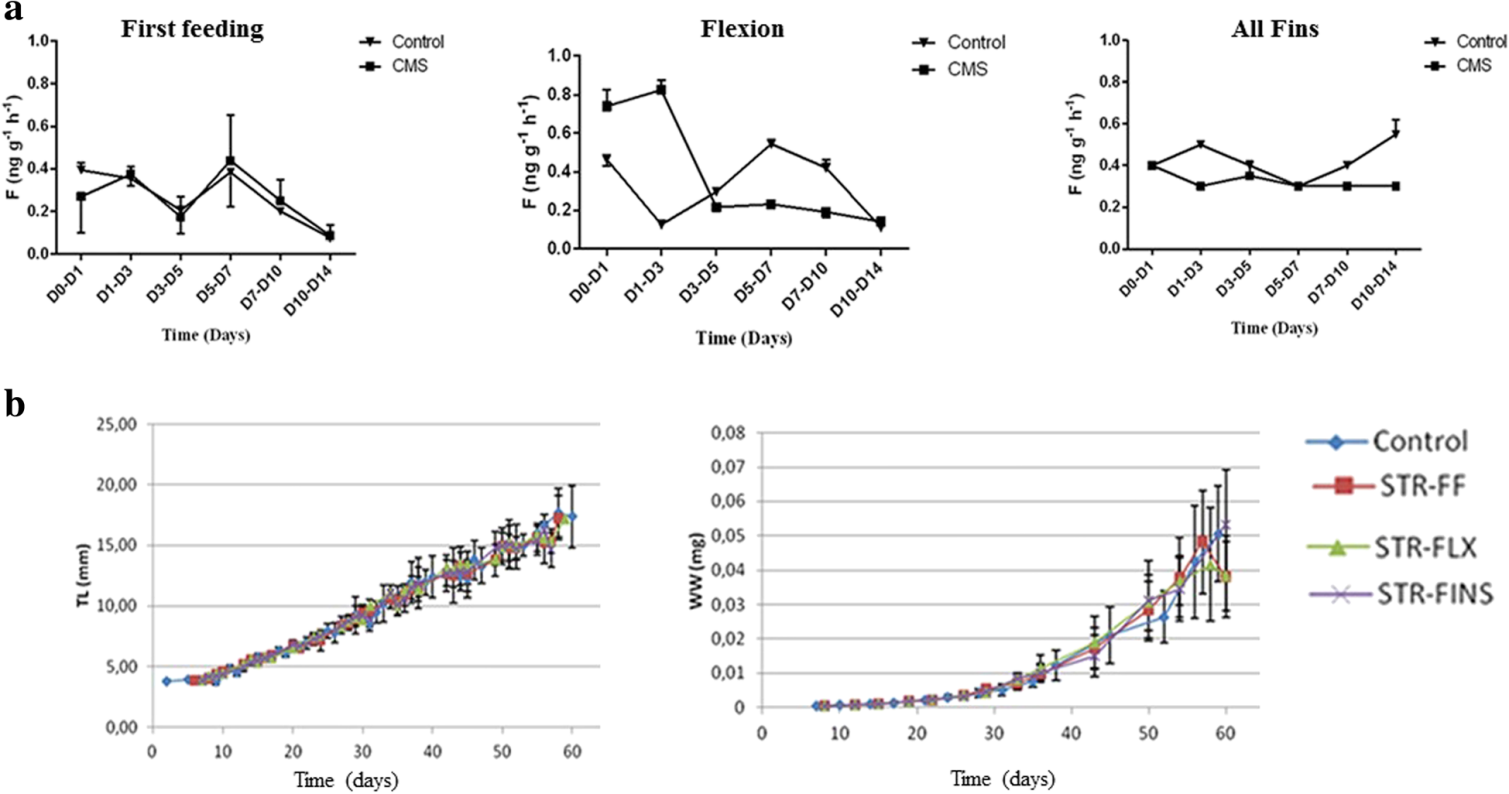
**a** Cortisol release rates in the holding tanks of *Phase 1*: The early-life protocol was applied at different periods, i.e. from first feeding to flexion (Group FF), from flexion to the formation of all fins (Group FLX) and from the formation of all fins to the full cover of body with melanophores (Group ALL FINS). One group remained undisturbed and served as control (CON). **b** Growth performance during *Phase 1*: Growth performance during (TL: total length; WW: wet weight) the period that the early-life protocol was applied (days 5 to 60 post hatching, *Phase 1*). Control: control larvae; STR-FF: larvae exposed to early-life events from first feeding onwards flexion; STR-FLX: from flexion onwards formation of all fins; STR-FINS: from the formation of all fins onwards full cover of body by melanophores. Values are given as mean ± S.D. (*n* = 10 per group and sampling point)

Water cortisol release rates showed no statistically significant differences in any of the groups where the experimental protocol was applied, compared to the respective controls (Fig. 1a). Values of the cortisol release rates for all groups were low and fluctuated between 0.05 and 0.86 ng g^−1^ h^−1^.

**Fig. 2:**
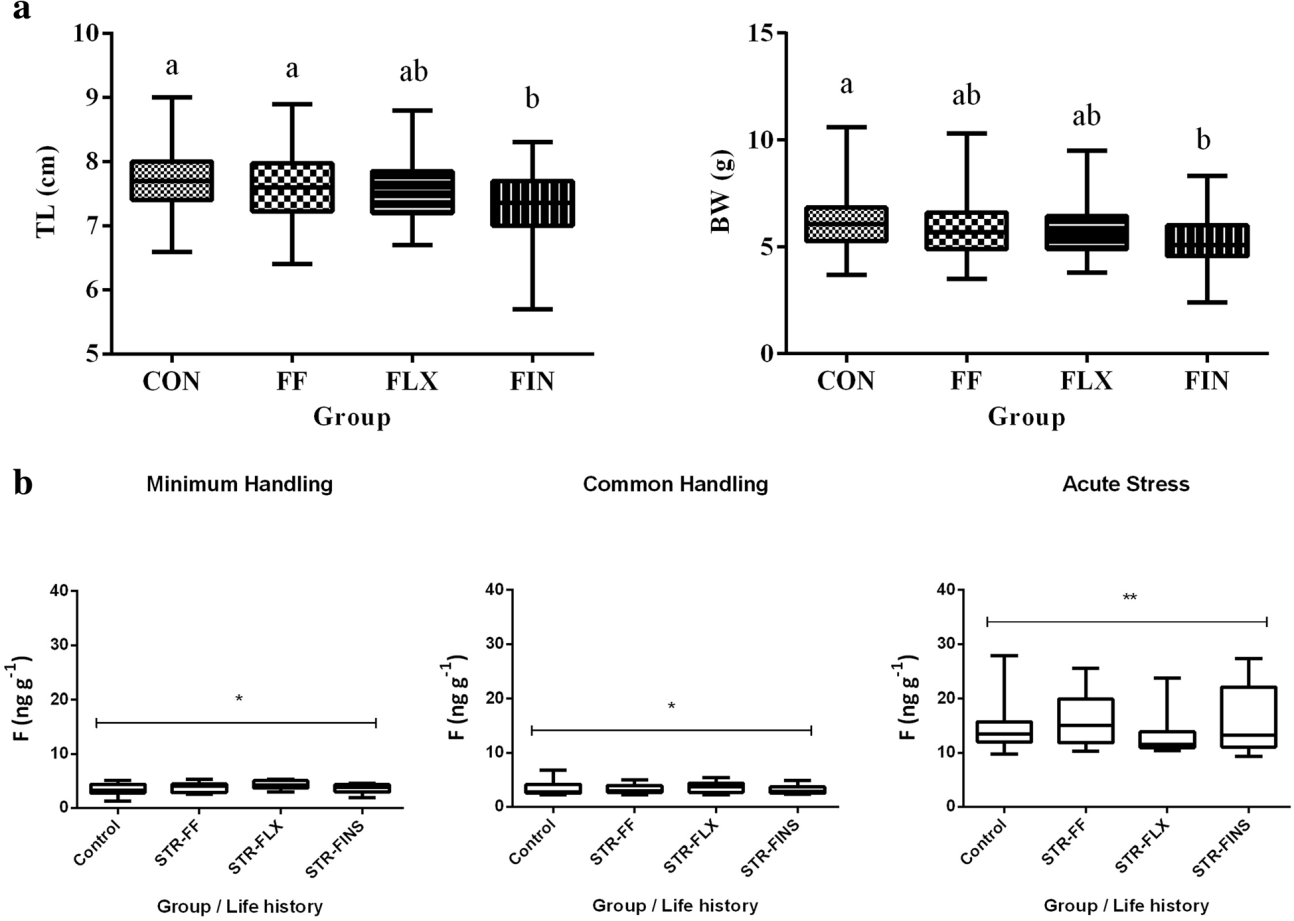
**a** Growth performance during *Phase 2* (Total Length, BodyWeight) of juvenile gilthead sea bream two months after the end of the early-life events. All fish were held under the same weaning and pre-growing rearing conditions. Groups are indicated according to their early larvae history. Box and whiskers (min to max) is the outcome of 20 measurements per group. **b** Whole body cortisol levels during *Phase 2*: Whole body cortisol levels (x ± S.D., n = 10) of juvenile gilthead sea bream according to early life stress (CON: Control; STR-FF, STR-FLX, STR-FINS: chronic mild stress for 14 days after the beginning of first feeding, flexion and the formation of all fins, respectively) and sampling method (minimum handling, common handling and 1 h post-acute stress)

The multiple linear regression analysis for both total length and wet weight showed that the protocol, had no significant effect on the growth rate of larvae at the end of the period that it was applied (until 60 dph) (Fig. 1b). However, differences in the mean total length or in the mean body weight among the experimental groups were observed at the end of *Phase 2*, i.e. two months after the end of *Phase 1* (juveniles up to the size of approximately 8.5 cm) with the group that had experienced early-life events at the stage of all fins (FINS) showing the lowest mean total length (7.3 ± 0.49 cm) and weight (5.3 ± 1.2 g) compared to the other three groups (control group: TL = 7.7 ± 0.5 cm, BW = 6.3 ± 1.5 g; FF group: TL = 7.6 ± 0.6 cm, BW = 6.0 ± 1.5 g; (FLX group: TL = 7.6 ± 0.6 cm, BW = 5.9 ± 1.5 g) (Fig. 2a).

### Acute stress response

Early life history did not affect whole-trunk cortisol concentrations of juvenile gilthead sea breams, either prior to or after exposure to acute stressors (Fig. 2b). In particular, minimum mean cortisol concentrations were found, regardless of the early life history, both in fish caught by minimum handling (1.3 to 5.4 ng g^−1^) or caught by common handling (2.2 to 6.9 ng g^−1^). However, in all groups statistically significant (*p* < 0.001) higher whole-body cortisol concentrations were found in acute stressed fish (9.3 to 27.9 ng g^−1^) compared to fish exposed only to minimum and common handling (Fig. 2b).

### Transcriptome sequencing

Illumina RNA sequencing resulted into a total of 633,216,227 assembled bases representing 580,011 transcripts. This dataset was used as reference transcriptome in order to map the reads from *Phase 1* (P1, early-life events) and reads from *Phase 2* (P2, acute stress applied to juveniles with and without early-life exposure events). In total 80–83 % of *Phase 1* reads and 69–74 % of *Phase 2* reads were successfully mapped onto the constructed reference transcriptome (Table 1). Raw sequence data have been deposited in the Short Read Archive (SRA) database of NCBI with the accession number: SRP062962.

**Table 1:** Overview of sequence assembly and mapping results

Total assembled bases:	633,216,227
Total trinity transcripts:	580,011
Percent GC content:	44.35
Median contig length:	471
Average contig:	1,092
Mapped reads Phase 1	80.74–83.23 %
Mapped reads Phase 2	69.62–73.32 %

### Differential expression

**Fig. 3:**
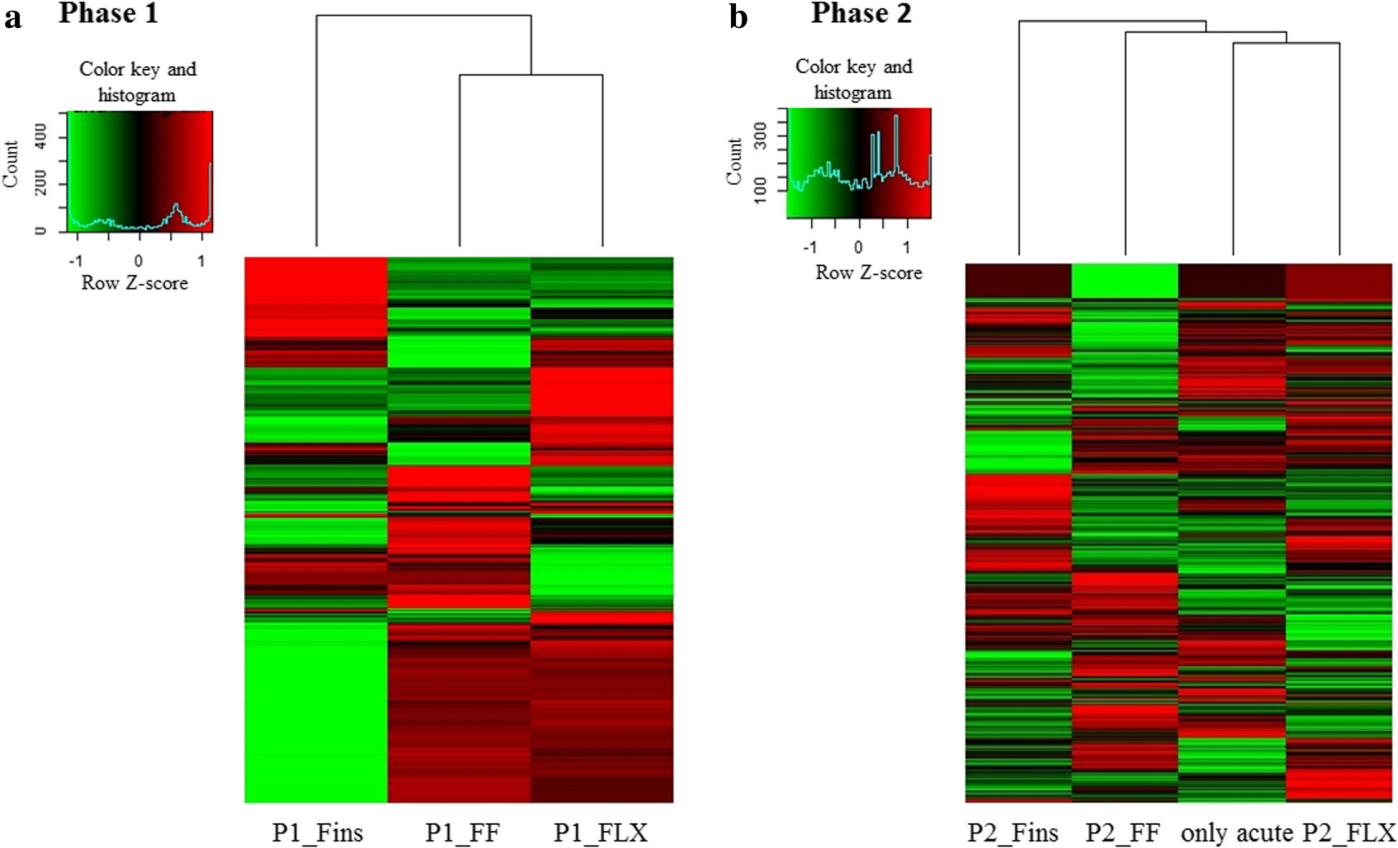
Heatmap analysis. **a** Heatmap of transcripts being either only up or down regulated during *Phase 1*. **b** Heatmap of transcripts being either only up or down regulated during *Phase 2.* Rows represent the log2-fold changes of different transcript expression levels and columns the experimental sampling points. The relative quantitative changes within the row are represented in color: red indicates a relative higher expression whereas green indicates a relative lower expression level

Three sets of putative differentially expressed transcripts were generated, with the first one containing transcripts with *p*-value ≤ 0.001, the second one with FDR < 0.05 and the third one with transcripts showing in all three biological replicates either only up or only down (no transcript found in any of the three replicates) regulation during stress. In Table 2 the amount of differentially expressed transcripts detected at each threshold is shown. The tendency of the transcripts counts identified as differentially expressed between samples was found to be similar in all three datasets. Downstream analysis was performed with all three datasets, but only the third set of transcripts is presented in the figures. Expression patterns using heat map analysis of transcripts differentially expressed during *Phase 1* showed that stage P1_FINS clusters separately from the other two stages (Fig. 3a). Heatmap analysis of significant differentially expressed transcripts during *Phase 2* of the experiment showed, that at stage “formation of all fins” (P2_FINS) the expression profile differs from the other two stages as well as from the expression profile of juveniles that have not experienced early life stress (Fig. 3b).

**Table 2:** Number of differentially expressed transcripts at three different significant tresholds

Experiment	*p-*value < 0.001	FDR < 0.05	Only up or down regulated
Phase 1			
FF control vs FF stress	1174	584	288
FLX control vs FLX stress	642	192	328
Fins control vs Fins stress	1027	507	576
Phase 2			
FF control vs FF stress	762	240	370
FLX control vs FLX stress	1234	685	619
Fins control vs Fins stress	670	197	332
Acute Stress			
Brain Control vs Brain Stress	812	272	384

### Cluster analysis

**Fig. 4:**
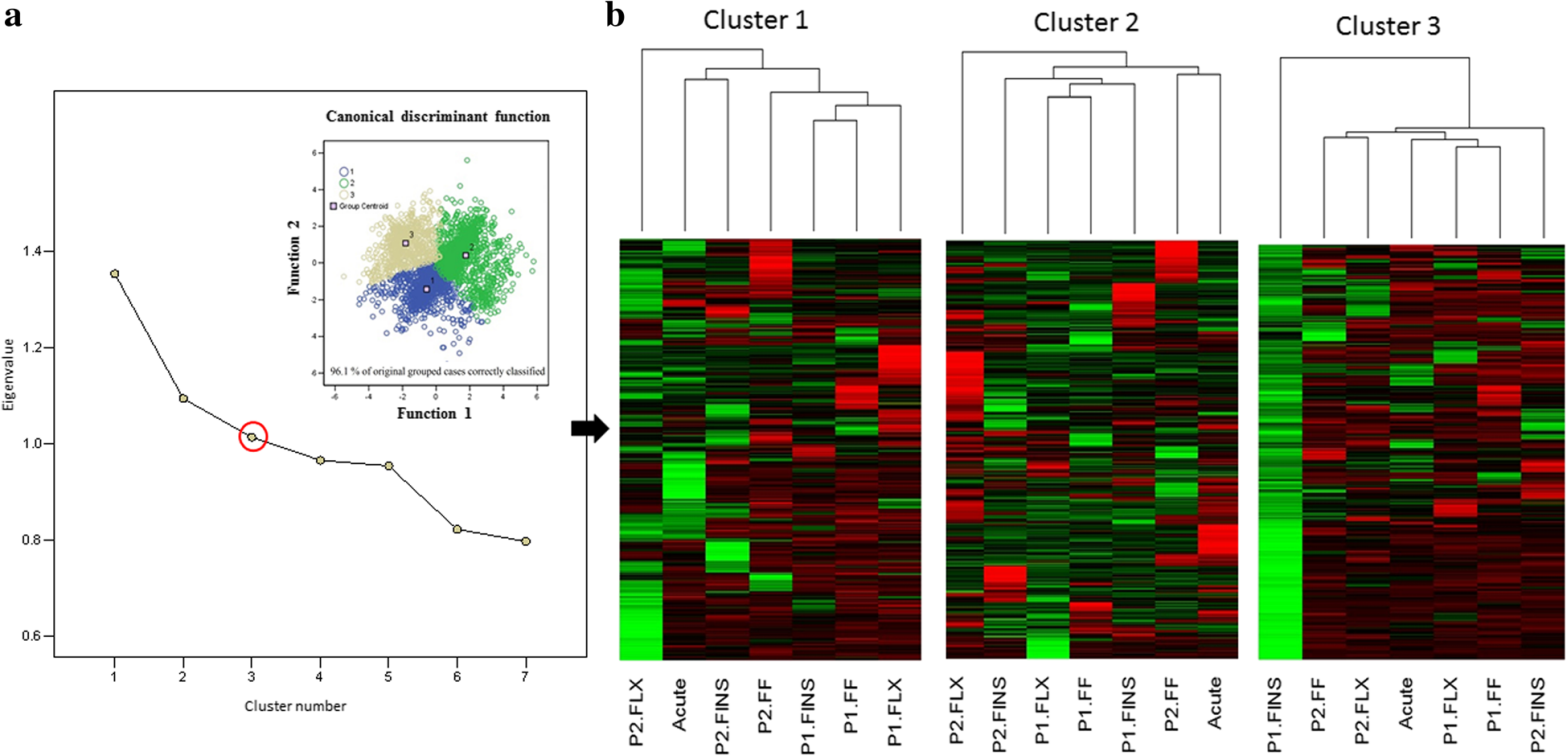
**a** Cluster analysis: Clustering of all (*Phase 1* and *Phase 2*) sequences being either up- or down regulated during stress are shown. The optimal number of clusters was obtained by generating a scree plot with the numbers of clusters on the x-axes and the distance between groups on the y-axes. The plot suggests 3 clusters as the best solution (red circle). Transcripts were further classified by the K means method. The data points given were grouped in three main clusters. The average value of the cases contained in each cluster is shown by the group centroid. Discriminant analysis correctly classified 96.1 % of the original grouped cases. **b** Heatmap analysis of the three clusters separately comprising all transcripts (*Phase 1* and *Phase 2*) either up- or down regulated. Rows represent the log2-fold expression changes of different transcripts and columns the experimental sampling points. The relative quantitative changes within the rows are represented in color: red indicates a relative higher expression whereas green indicates a relative lower expression level

**Fig. 5:**
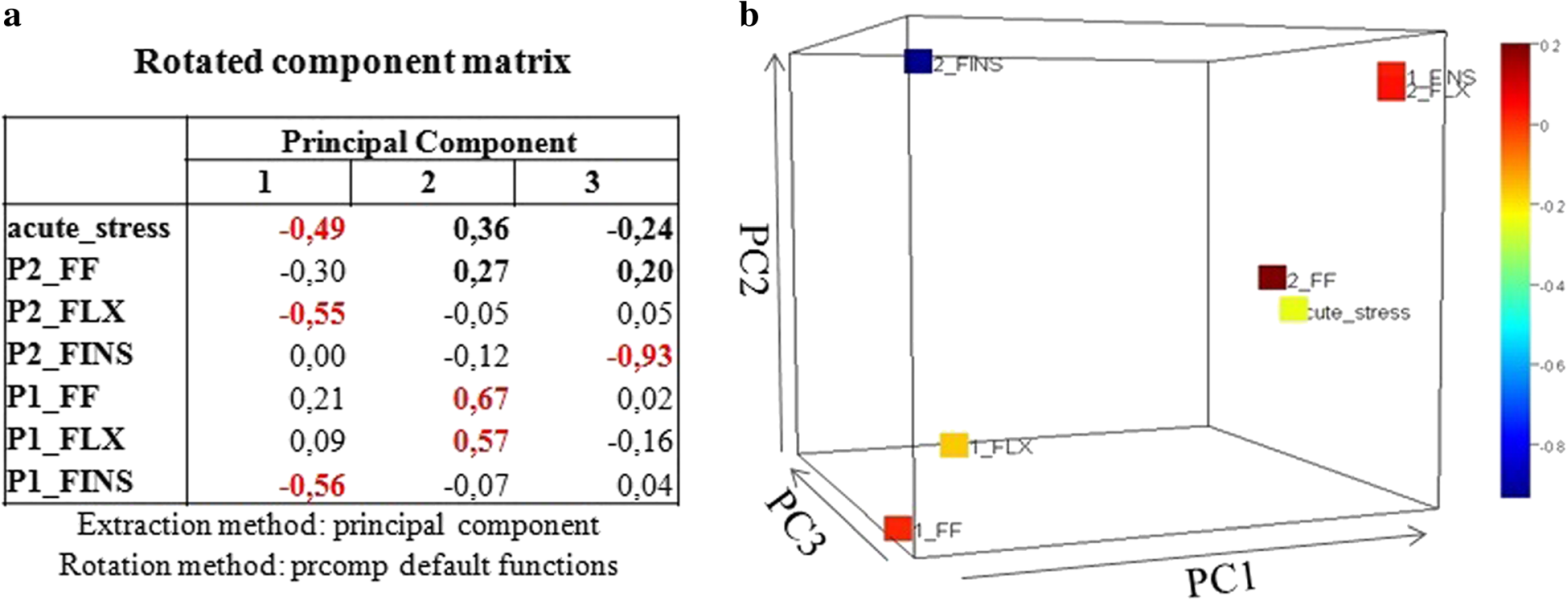
Principal Component Analysis (PCA). **a** Rotated component matrix. Values greater than or close to 0.5 are in red. **b** PCA of rotated values. All seven experimental time points are projected in a 3-dimensional plane. Color code reflects their corresponding values

Cluster analysis methods were performed with the three sets of all determined differentially expressed transcripts (*Phase 1* and *Phase 2*) resulting in similar results. The third and most stringent dataset (transcripts showing only up- or down-regulation) is presented. Hierarchical clustering and the subsequently generated scree plot (Fig. 4a) as well as cluster heatmap analysis (data not shown) suggested three clusters to be most appropriate. K-means clustering classified transcripts into each cluster. Classification was corroborated by discriminant analysis, grouping 96.1 % of original grouped cases correctly to the predetermined clusters (Fig. 4a). Heatmap analysis of the predefined clusters is shown in Fig. 4b with down-regulated transcripts during P2-FLX in cluster 1, mostly up-regulated transcripts during P2-FLX in cluster 2 and down-regulated transcripts during P1-FINS in cluster 3 (Blastx matches of transcripts in Additional file 2). For further structure detection within the data, PCA analysis was applied. PCA analysis revealed a distinct transcript pattern in the dataset with the first three factors having eigenvalues greater than 1 explaining 49 % of the variability. This is in concordance with the generated scree plot (Fig. 4a). The rotated factor matrix shows that P2-FINS is associated with the third factor whereas the second is highly correlated to P1-FF and P1-FLX and the first to P2-FLX, P1-FINS as well as acute stress (Fig. 5a). However, the transcription pattern of acute stress clusters with those of P2-FF due to the values of factor 2 and 3 (Fig. 5b, in bold) whereas P2-FLX and P1-FINS are not particularly correlated to the other two factors. Larvae exposed to the experimental protocol at stages FF and FLX have similar expression patterns, whilst stage P2-FINS differs from all other cases.

### Annotation

Blastx search against the NCBI database successfully assigned 4451 out of 5258 differentially expressed transcripts to a putative protein. Within the GO categorization Molecular function, 22 % of the transcripts were classified to the GO term ATP binding. Within the GO categorization Biological Process, 18 % were classified to the GO term proteolysis and 17 % to the GO term oxidation-reduction process (Additional file 3: Figure S2 and Additional file 4: Figure S3).

### GO:000695: stress response

**Fig. 6:**
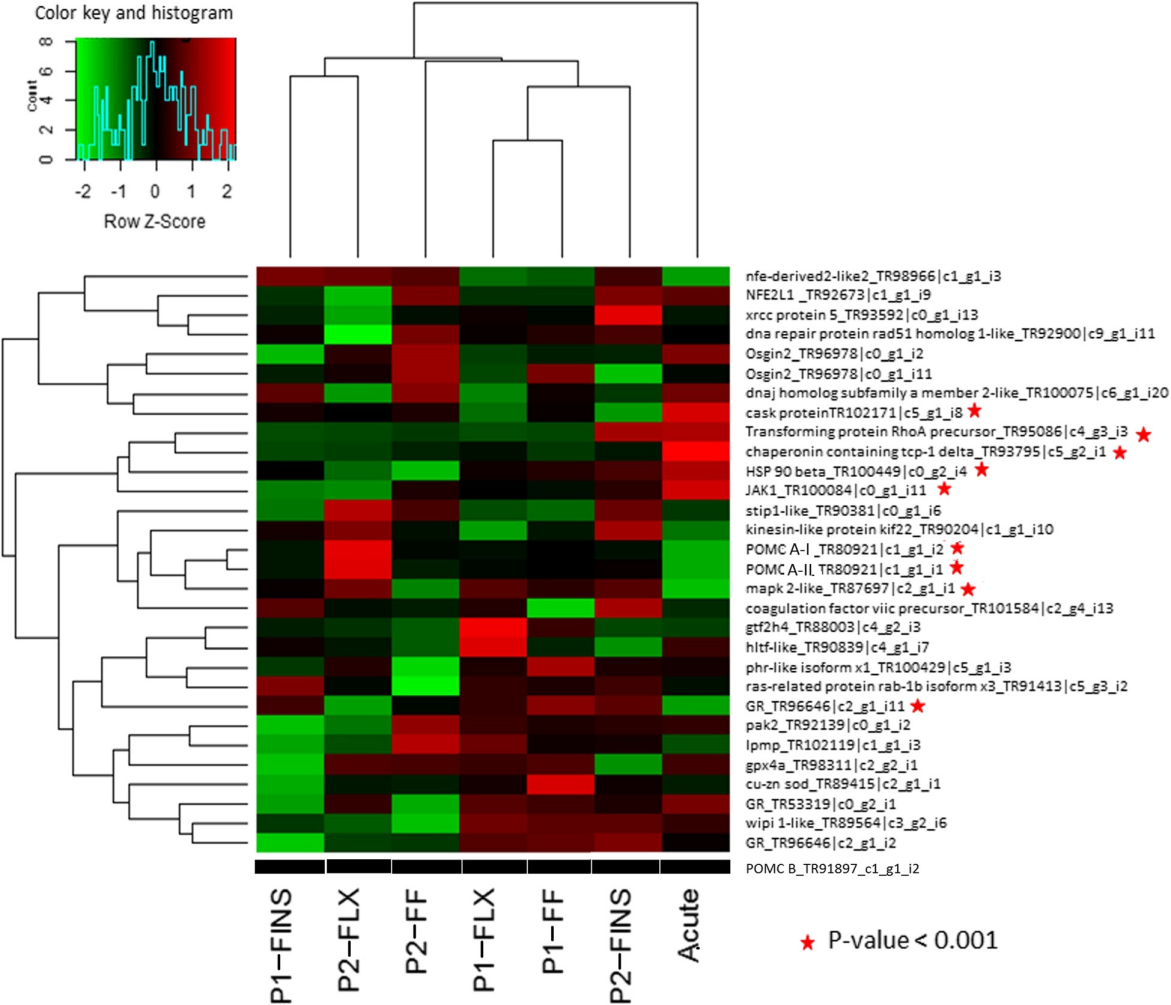
Gene ontology stress response: Heatmap analysis of all transcripts categorized into the GO group “GO:000695: stress response”. Rows represent the log2-fold changes of transcripts expression and columns the experimental sampling points. The relative quantitative changes within the row are represented in color: red indicates a relative higher expression whereas green indicates to a relative lower expression level. *P* values < 0.001 of the transcripts studied during acute stress are indicated by an asterisk

Transcripts classified to the GO category “stress response” were isolated and their expression profile determined. Transcript expression patterns obtained from the acute stress experiment clearly differed from the other six experimental groups under study (Fig. 6).

## Discussion

In order to investigate the effect of exposure to early-life events on development and performance of gilthead sea bream, a stress protocol consisting of two different optical, mechanical or social stimuli was applied randomly on a daily basis for a period of two weeks starting at the beginning of three selected developmental stages (first feeding, flexion and development of all fins). The experimental protocol was based on an unpredictable chronic low intensity stress (UCLIS) protocol, previously developed for the European sea bass [24]. In contrast to the results obtained for the European sea bass, the particular protocol did not affect survival, larvae performance and water cortisol release rates during the *Phase 1* of the experiment (Fig. 1a, b). In addition, remarkable lower basal cortisol release rates were observed in the present study compared to those of the European sea bass [24]. These differences in the effects of the applied stressors among the two species were expected as a previous comparative study on European sea bass and gilthead sea bream showed that they possess different stress responsiveness [8]. A significant difference was observed regarding skeletal deformities within the flexion group, where a higher percentage of individuals were detected with improperly developed operculum and with deformed spinal cord (Table 3).

**Table 3:** Percentage of individuals within the flexion group with skeletal deformities

	FF	FLX	FINS	C
operculum	0 %	6 %	0 %	0 %
skeletal	6 %	22 %	3 %	3 %
no swim bladder	0 %	0 %	3 %	3 %

Concerning *Phase 2*, statistically significant differences in total length as well as in body weight for those juveniles that experienced early-life events during the FIN stages were evident (Fig. 2a). However, no effect in whole-trunk cortisol concentrations of juvenile fish was detected, neither prior to nor after exposure to acute stressors (Fig. 2b). Higher whole-trunk cortisol concentrations were only found in acute stressed fish compared to fish exposed to minimum and common handling (Fig. 2b).

Taken together, during *Phase 1* the experimental protocol had no effect on water-born cortisol concentrations and on the growth performance of gilthead sea bream larvae. This is in contrast with the case of European sea bass, where first feeding and flexion stages appeared to be more sensitive to the stimuli applied. It therefore appears that the gilthead sea bream is more tolerant than European sea bass to ordinary husbandry and managerial practices during early ontogeny. It has to be noted here, that although water renewal that gradually increased was an improvable parameter, measurements of water-born cortisol concentration in general are more appropriate to be performed in running water (of a given water renewal rate) than in static water [28, 29].

During *Phase 2,* similar results were observed for both species, as fish, that had experienced an early-life event during the stage of the formation of all fins showed the worst performance. These results indicate that the early-life protocol applied, which is characterized by the unpredictability, variety and moderate intensity of the applied stimuli, provides a relative realistic model to evaluate the impact of daily aquaculture practices on fish performance. In addition, it can be used as a tool to investigate the impact of early-life events and of genome-environmental interactions on important life-history traits and stress-coping strategies at subsequent stages of development, in vertebrates with no perinatal maternal distress and complex parental care behavior.

With the onset of NGS technologies, transcriptomic information and global gene expression patterns of any organism of interest can be assessed, paving the way to investigate in entire transcriptome changes in relation to specific conditions, treatments or tissues. However, for robust gene expression pattern results, high throughput data are necessary as well as biological replicates. In the present study, three biological replicates from each condition were submitted to high throughput Illumina paired-end sequencing, and strict thresholds were set resulting in a robust data set. To assess differential expression, a reference transcriptome was first constructed comprising a total of 580,011 trinity transcripts (Table 1) with an average length of 1,092 bp. Subsequently paired-end reads of experimental *Phase 1* as well as of *Phase 2* were mapped onto the constructed reference transcriptome (up to ~83 % and up to ~ 74 % respectively, Table 1). RNAseq enables also the detection of multiple transcript variants and thus does not reflect the number of genes of an organism. For further downstream data analysis three dataset with different threshold values were constructed. The fact that all three datasets show the same downstream expression patterns points to the robustness and the reliability of the results obtained*.*

While cortisol measurements as well as total length and body weight did not show any effect when larvae were subjected to the early-life protocol (*Phase 1*), transcriptome analysis revealed differentially expressed transcripts with the developmental stage “all fins” (P1-FINS) separating itself from the other two (Fig. 3a). These results point to the fact that the most diverged phase in term of gene expression pattern during development seems to be at the phase of the development of all fins, which also has an impact later on in development and in particular in juveniles as shown in the expression patterns of *Phase 2.* Obtained expression patterns here showed, that juvenile fish experienced early-life events during the formation of fins (P2_FINS) differ from the other two early-life events (P2_FF and P2_FLX) as well as from the samples without any early-life event (Fig. 3b). This underlines once more the importance of the fin formation stage during the development of the gilthead sea bream.

**Fig. 7:**
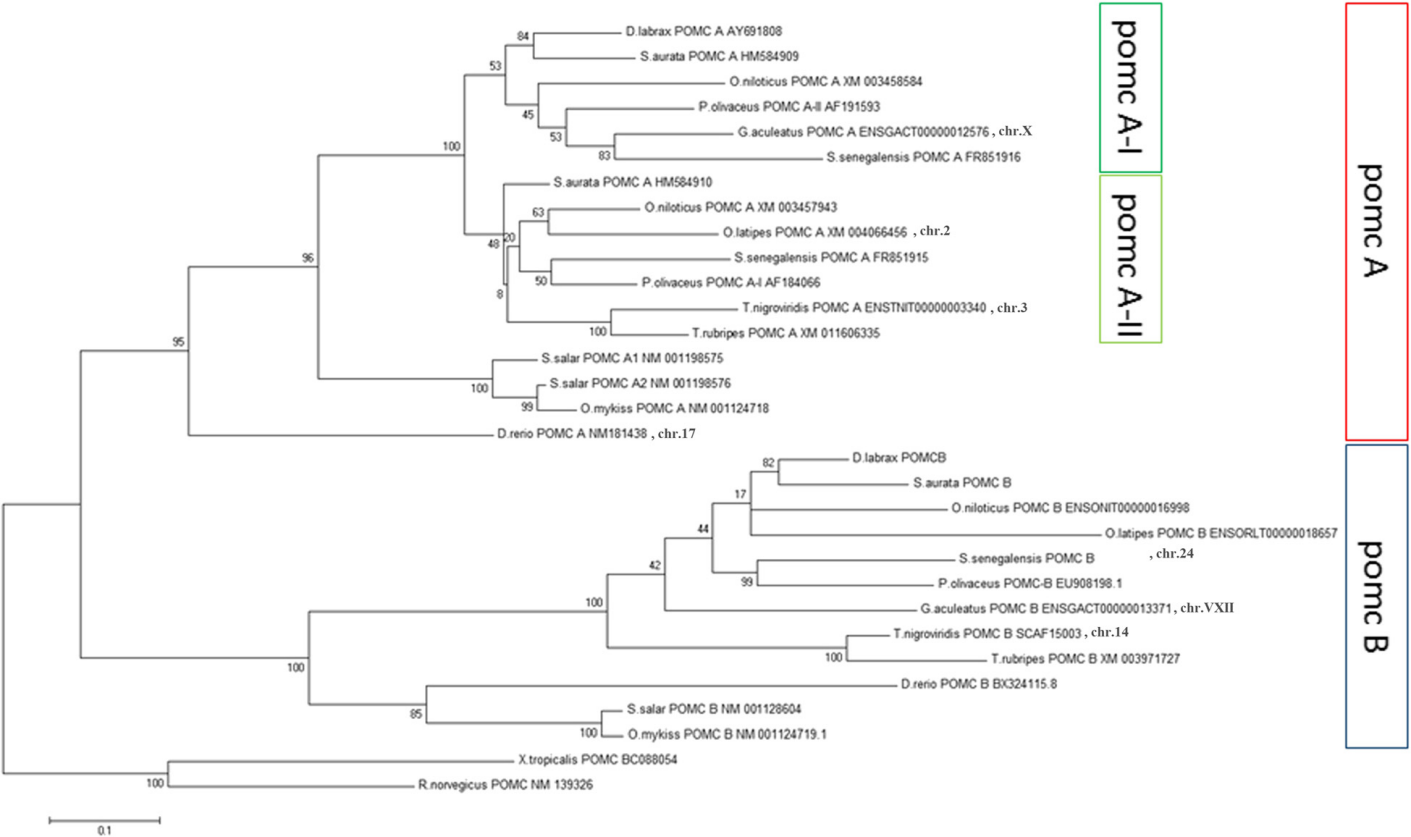
Gene tree of the paralogous gene *pomc*. Molecular Phylogenetic analysis conducted in MEGA5 [42] using Maximum Likelihood method based on the Tamura-Nei model [43]. The analysis involved 31 nucleotide sequences encoding for propiomelanocortin (*pomc*) retrieved from GenBank in a total of 14 species. The tree is drawn to scale, with branch lengths measured in the number of substitutions per site and clearly separates *pomc* paralogs into two main groups, *pomc a* and *pomc b. Pomc a* comprises two subgroups *pomc a-I* and *pomc a-II*. For the gilthead sea bream *pomc-b* was identified in the present study (transcript id: TR91897|c1_g1_i2)

**Fig. 8:**
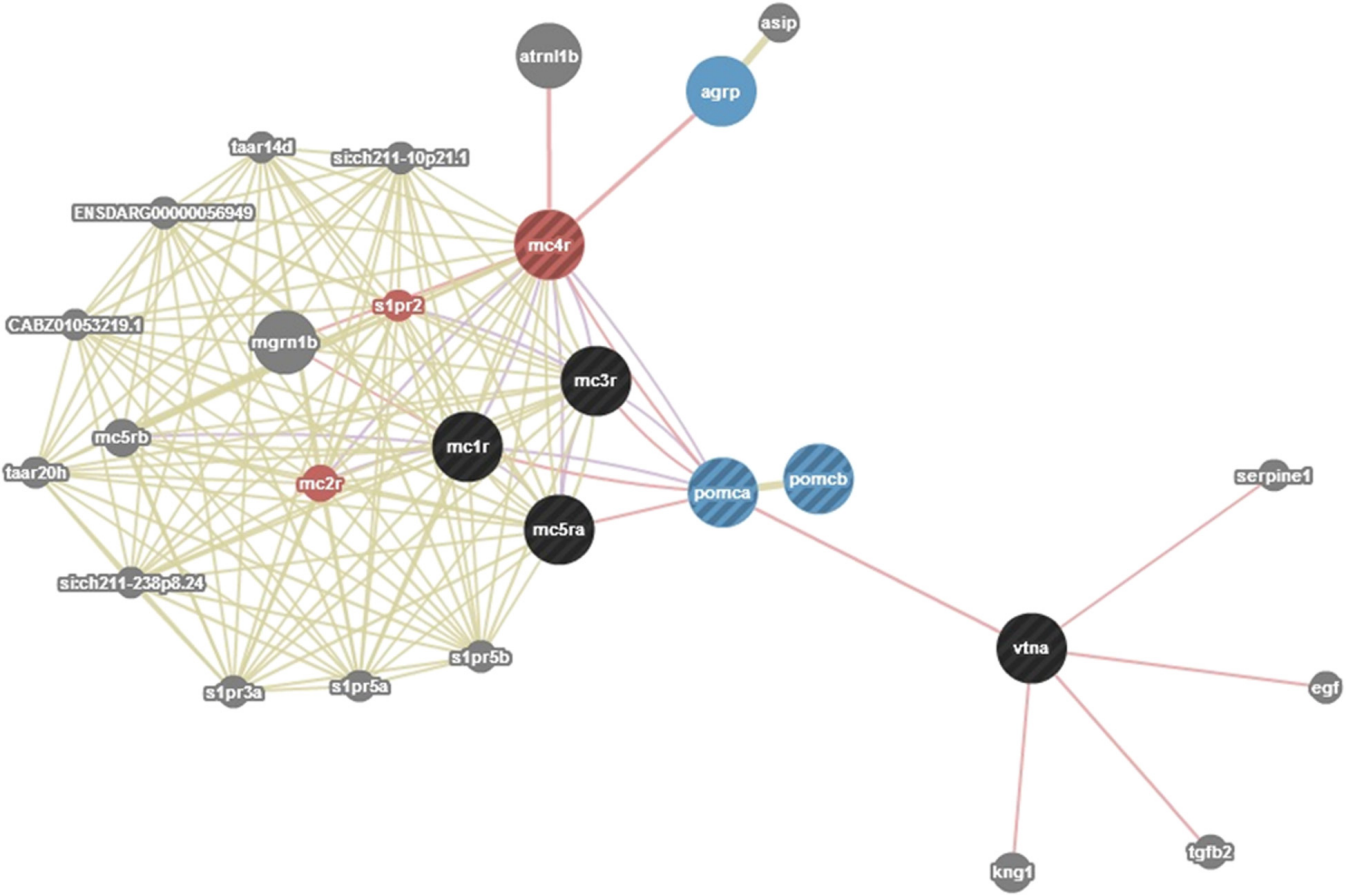
Gene network. Gene network showing the interaction of the orthologous genes *pomc-a* and *pomc-b* obtained by GeneMANIA. Genes having physiological interactions either with *pomc-a* or *pomc-b* were used to create GeneMania network. Input genes are indicated with stripes and are either of black, red or blue color. Intermediate genes added by the tool are illustrated in grey circles. Colored genes denote functions associated by the tools: blue = “G-protein coupled receptor signaling pathway”; red = “G-protein coupled peptide receptor activity”. Edge colors indicate the type of interaction: shared protein domains (green), co-expressed genes (violet) and physical interactions (pink) are shown

In order to further investigate gene expression patterns in relation to stress response, expression values of all significantly expressed transcripts obtained from *Phase 1* and *Phase 2* were submitted to cluster analysis, using hierarchical clustering, k-means clustering, and PCA analysis. In general, the K-means clustering method is recommended when the population of genes to analyze is more than 200 [38]. To define the number of clusters, hierarchical cluster analysis was performed and agglomeration values were plotted on a scree diagram (Fig. 4a). Following the elbow rule the cluster number was determined to be three. The analysis was repeated using K-means analysis with the number of cluster set to three. Cluster membership was corroborated by further discriminant analysis classifying correctly 96.1 % of original grouped cases (Fig. 4a). Further PCA analysis (Fig. 5) showed that transcripts belonging to P1-FINS and P2-FLX were highly correlated to each other. The group belonging to the “acute stress” experiment correlates at PC1 together with P1-FINS and P2-FLX, but differs from them at PC2 and 3. Thus, this may indicate that on the one hand larvae exposed to early-life events during the formation of fins (P1-FINS) have a similar response to stress as juveniles exposed only to acute stress. On the other hand when larvae are exposed to early-life events during the flexion stage, juveniles do not responds much differently than those juveniles exposed only to acute stress (P2-FLX). The only group being close to “acute stress” in all three components is P2-FF, suggesting that the early-life protocol applied during first feeding of the fish has no effect at all on the later life history. P1-FLX and P1-FF are correlated to the second component reflecting similar reaction to early life events either at the first feeding stage or at the stage of flexion. Thus concerning *Phase 1,* again the stage “all fins” is the most diverged one. Concerning *Phase 2,* only when the early-life protocol is applied during the “all fins” stage it has an impact on juveniles. Such stress response revealed in the transcriptome data, presumably reflects impacts of handling upon fish wellbeing, as seen in the growth performance in gilthead sea bream juveniles (Fig. 2a). Based on these results, it appears that the molecular response to early-life events (*Phase 1*) is similar when applied at stages FF and FLX, whereas if applied at stage FINS, a different expression pattern is seen. Similar expression patterns were detected for the stages FINS and FLX during *Phase 1* and *Phase 2* respectively. Juveniles exposed to acute stress displayed the most altered expression patterns when they have been exposed to early-life events during the formation of fins (P2-FINS). In contrast, comparable patterns in gene expression were detected in juveniles that had experienced early-life events at the very first larval stage (P2-FF). These results can have implications for the development of aquaculture practices and determine which are the least sensitive stages for conducting handling procedures. To further investigate stress response related genes, expression patterns of transcripts classified after GO ontology to the GO group “stress response” are shown in Fig. 6. In total, nine transcripts showed a *p*-value < 0.001 during the experiment applying acute stress to juveniles without having experienced early-life events (acute). Notably, P1-FF, P1-FLX and P2-FINS again form a group with the latter one separating itself from the former two as in the PCA plot of significantly expressed transcripts. Concerning P1-FINS and P2-FLX they cluster again together in one group, whereas P2-FF clusters to the group comprising P1-FF, P1-FLX and P2-FINS. The expression pattern of acute stress does not cluster to any other group. Looking at the expression pattern illustrated in Fig. 6, it is of interest to pinpoint the orthologous pair of genes annotated by NCBI blastn as proopiomelanocortin, *pomc-a* (HM584909) and *pomc-b* (HM584910). *Pomc-a* orthologs and their differential expression in a tissue panel was first described by Cardoso et al.[39]. In contrast to the information available at NCBI, Cardoso et al. [39] assigned the accession numbers HM584909 and HM584910 to *pomc-aI* and *pomc-aII*. The paralogous gene to *pomc-a, pomc-b* in the gilthead sea bream was identified for the first time in the present study (transcript id: TR91897|c1_g1_i2), without, however, significant differential expression in any of the stress experiments. The identification of gilthead sea bream *pomc-b* was evaluated by comparative mapping and phylogenetic analysis, which confirmed the identification of the gilthead sea bream *pomc-b* paralog (Fig. 7). However only *pomc-a* paralogs showed significant differentially expression, which may explain why the *pomc-b* paralog was not identified until now (Fig. 6). Gene network analysis by GeneMANIA [40] shows physical interactions with melanocortin receptors, vitronectin, agouti related protein, agouti signaling protein and attractin-like 1b only for *pomc-a* and not for *pomc-b* (Fig. 8)*.* This also supports the hypothesis that only the paralogs *pomc-aI* and *pomc-aII* respond to stress (Fig. 6) and *pomc-b* most probably underwent sub- or neofunctionalization and thus is involved in pathways other than stress response. Tissue specific expression analysis of the three pomc homologs in African cichlid fishes showed that only the whole brain and embryonic tissue expressed all paralogs of *pomc,* which explains why *pomc-b* was identified in the present study. In addition, it shows that some tissues express only the one of the paralogs (e.g. only *pomc –b* was detected in skin*)*, which also pinpoints to a sub- or neofunctionalization [41].

## Conclusions

The early-life stress protocol appears to be useful to investigate interactions between gene expression patterns and environmental factors during early life. Gene expression patterns appeared to be more subtle than physiological measurements to detect response in the gilthead sea bream after early-life exposure to mild stimuli, as well as after acute stress. RNAseq analyses further showed the robustness of the experimental set up, and detected distinct expression patterns according to the time point of early-life events during development. It further revealed that juvenile fish are sensitive to the timing of exposure to the early-life protocol during larval development. Based on the data obtained in the present work it can be concluded that applying ordinary mild stimuli very early in development (at first feeding) does not affect performance nor the acute stress response at juvenile stage, whereas when the same events are applied at the phase of flexion or of formation of all fins, the stress response varies with the formation of all fins being the most critical stage during development.

## Additional files

Additional file 1: Experimental set up. Illustration of the experimental set up used in the present study. Red bars indicate the time range where the early-life protocol were applied. FF: first feeding, FLX: first appearance of flexion Fins: first appearance of fins. (JPG 70 kb) Additional file 2: Blastx matches of transcripts classified in cluster 1, 2 or 3. Blastx results of transcripts in cluster 1, 2, and 3 separately. (XLSX 145 kb) Additional file 3: GO category “Molecular function” of identified transcripts. Summary of GO category “Molecular function” of transcripts significantly expressed in any of the sampling points. (JPEG 81 kb) Additional file 4: GO category “Biological process” of identified transcripts. Summary of GO category “Biological process” of transcripts significantly expressed in any of the sampling points. (JPEG 73 kb)

## References

[1] Stott GH (1981). What is animal stress and how is it measured?. J Anim Sci.

[2] Vera LM, Montoya A, Pujante IM, Pérez-Sánchez J, Calduch-Giner JA MJM, Moliner JS-VFJ (2014). Acute stress response in gilthead sea bream (*Sparus aurata* L.) is time-of-day dependent: Physiological and oxidative stress indicators. Chronobiol Int.

[3] De Santis C, Bartie KL, Olsen RE, Taggart JBTDR (2015). Nutrigenomic profiling of transcriptional processes affected in liver and distal intestine in response to a soybean meal-induced nutritional stress in Atlantic salmon (*Salmo salar*). Comp Biochem Physiol Part D Genomics Proteomics.

[4] Schreck CB, Contreras-Sanchez W, Fitzpatrick MS (2001). Effects of stress on fish reproduction, gamete quality, and progeny. Aquaculture.

[5] Aluru N, Vijayan MM (2009). Stress transcriptomics in fish: A role for genomic cortisol signaling. Gen Comp Endocrinol.

[6] Barton BA, Iwama GK (1991). Physiological changes in fish from stress in aquaculture with emphasis on the response and effects of corticosteroids. Annu Rev Fish Dis.

[7] Wendelaar Bonga SE (1997). The stress response in fish. Physiol Rev.

[8] Fanouraki E, Mylonas CC, Papandroulakis N, Pavlidis M (2011). Species specificity in the magnitude and duration of the acute stress response in Mediterranean marine fish in culture. Gen Comp Endocrinol.

[9] Schreck CB (1981). Stress and Fish.

[10] Schreck CB (1982). Stress and rearing of salmonids. Aquaculture.

[11] McCormick SD, Shrimpton JM, Carey JB, O&apos;Dea MF, Sloan KE, Moriyama S, Björnsson BT (1998). Repeated acute stress reduces growth rate of Atlantic salmon parr and alters plasma levels of growth hormone, insulin-like growth factor I and cortisol. Aquaculture.

[12] Korte SM, Olivier B, Koolhaas JM (2007). A new animal welfare concept based on allostasis. Physiol Behav.

[13] Tsalafouta A, Papandroulakis N, Gorissen M, Katharios P, Flik G, Pavlidis M (2014). Ontogenesis of the HPI axis and molecular regulation of the cortisol stress response during early development in *Dicentrarchus labrax*. Sci Rep.

[14] Teles M, Boltaña S, Reyes-López F, Santos MA, Mackenzie S, Tort L (2013). Effects of Chronic Cortisol Administration on Global Expression of GR and the Liver Transcriptome in *Sparus aurata*. Mar Biotechnol.

[15] Nesan D, Vijayan MM (2013). Role of glucocorticoid in developmental programming: Evidence from zebrafish. Gen Comp Endocrinol.

[16] Alsop D, Vijayan MM (2009). Molecular programming of the corticosteroid stress axis during zebrafish development. CompBiochemistPhysiol.

[17] Krasnov A, Koskinen H, Pehkonen P, Rexroad CE, Afanasyev S, Mölsä H (2005). Gene expression in the brain and kidney of rainbow trout in response to handling stress. BMC Genomics.

[18] Aluru N, Vijayan MM (2007). Hepatic transcriptome response to glucocorticoid receptor activation in rainbow trout. Physiol Genomics.

[19] Wiseman S, Osachoff H, Bassett E, Malhotra J, Bruno J, VanAggelen G, Mommsen TP, Vijayan MM (2007). Gene expression pattern in the liver during recovery from an acute stressor in rainbow trout. Comp Biochem Physiol - Part D Genomics Proteomics.

[20] Pittman K, Yúfera M, Pavlidis M, Geffen AJ, Koven W, Ribeiro L, Zambonino-Infante JL, Tandler A (2013). Fantastically plastic: Fish larvae equipped for a new world. Rev Aquac.

[21] Sarropoulou E, Kotoulas G, Power DM, Geisler R (2005). Gene expression profiling of gilthead sea bream during early development and detection of stress-related genes by the application of cDNA microarray technology. Physiol Genomics.

[22] Mininni AN, Milan M, Ferraresso S, Petochi T, Di Marco P, Marino G, Livi S, Romualdi C, Bargelloni L, Patarnello T (2014). Liver transcriptome analysis in gilthead sea bream upon exposure to low temperature. BMC Genomics.

[23] Calduch-Giner JA, Davey G, Saera-Vila A, Houeix B, Talbot A, Prunet P, Cairns MT, Pérez-Sánchez J (2010). Use of microarray technology to assess the time course of liver stress response after confinement exposure in gilthead sea bream (*Sparus aurata* L.). BMC Genomics.

[24] Tsalafouta A, Papandroulakis N, Pavlidis M (2015). Early life stress and effects at subsequent stages of development in European sea bass *(D. labrax*). Aquaculture.

[25] de Jesus EG, Hirano T, Inui Y, de Jesus EG (1991). Changes in cortisol and thyroid hormone concentrations during early development and metamorphosis in the Japanese flounder, *Paralichthys olivaceus*. Gen Comp Endocrinol.

[26] Pavlidis M, Karantzali E, Fanouraki E, Barsakis C, Kollias S, Papandroulakis N (2011). Onset of the primary stress in European sea bass *Dicentrarhus labrax*, as indicated by whole body cortisol in relation to glucocorticoid receptor during early development. Aquaculture.

[27] Ellis T, James JD, Stewart C, Scott A (2004). P: A non-invasive stress assay based upon measurement of free cortisol released into the water by rainbow trout. J Fish Biol.

[28] Adams S, Breck J, Schreck CB, Moyle PBE (1990). Bioenergetics. Methods for fish biology.

[29] Fanouraki E, Papandroulakis N, Ellis T, Mylonas CC, Scott AP, Pavlidis M (2008). Water cortisol is a reliable indicator of stress in European sea bass, *Dicentrarchus labrax*. Behaviour.

[30] Bolger AM, Lohse M, Usadel B (2014). Trimmomatic: A flexible trimmer for Illumina sequence data. Bioinformatics.

[31] Grabherr MG, Haas BJ, Yassour M, Levin JZ, Thompson DA, Amit I, Adiconis X, Fan L, Raychowdhury R, Zeng Q, Chen Z, Mauceli E, Hacohen N, Gnirke A, Rhind N, di Palma F, Birren BW, Nusbaum C, Lindblad-Toh K, Friedman N, Regev A (2011). Full-length transcriptome assembly from RNA-Seq data without a reference genome. Nat Biotechnol.

[32] Langmead B, Salzberg SL (2012). Fast gapped-read alignment with Bowtie 2. Nat Methods.

[33] Langmead B, Trapnell C, Pop M, Salzberg SL (2009). Ultrafast and memory-efficient alignment of short DNA sequences to the human genome. Genome Biol.

[34] Li B, Dewey CN (2011). RSEM: accurate transcript quantification from RNA-Seq data with or without a reference genome. BMC Bioinformatics.

[35] Robinson M, Mccarthy D, Chen Y, Smyth GK (2011). edgeR : differential expression analysis of digital gene expression data User’ s Guide. Most.

[36] Altschul SF, Gish W, Miller W, Myers EW, Lipman DJ (1990). Basic local alignment search tool. J Mol Biol.

[37] Conesa A, Götz S (2008). Blast2GO: A Comprehensive Suite for Functional Analysis in Plant Genomics. Int J Plant Genomics.

[38] Hair F, Taham R, REA, Black WCJ. Multivariate data analysis. Fith Ed. Prentice-Hall Int. Upper Saddle River: Pearson Prentice Hall; 1998.

[39] Cardoso JCR, Laiz-Carrion R, Louro B, Silva N, Canario AVM, Mancera JM, Power DM (2011). Divergence of duplicate POMC genes in gilthead sea bream *Sparus auratus*. Gen Comp Endocrinol.

[40] Zuberi K, Franz M, Rodriguez H, Montojo J, Lopes CT, Bader GD, Morris Q (2013). GeneMANIA prediction server 2013 update. Nucleic Acids Res.

[41] Harris RM, Dijkstra PD, Hofmann HA (2014). Complex structural and regulatory evolution of the pro-opiomelanocortin gene family. Gen Comp Endocrinol.

[42] Tamura K, Peterson D, Peterson N, Stecher G, Nei M, Kumar S (2011). MEGA5: Molecular evolutionary genetics analysis using maximum likelihood, evolutionary distance, and maximum parsimony methods. Mol Biol Evol.

[43] Tamura K, Nei M (1993). Estimation of the number of nucleotide substitutions in the control region of mitochondrial DNA in humans and chimpanzees. Mol Biol Evol.

