# Supplementary figures and images for: Transcriptomic changes in relation to early-life events in the gilthead sea bream (Sparus aurata)
Source: BMC Genomics. 2016 Jul 26;17:506. doi: 10.1186/s12864-016-2874-0 (PMC4962366; doi:10.1186/s12864-016-2874-0)

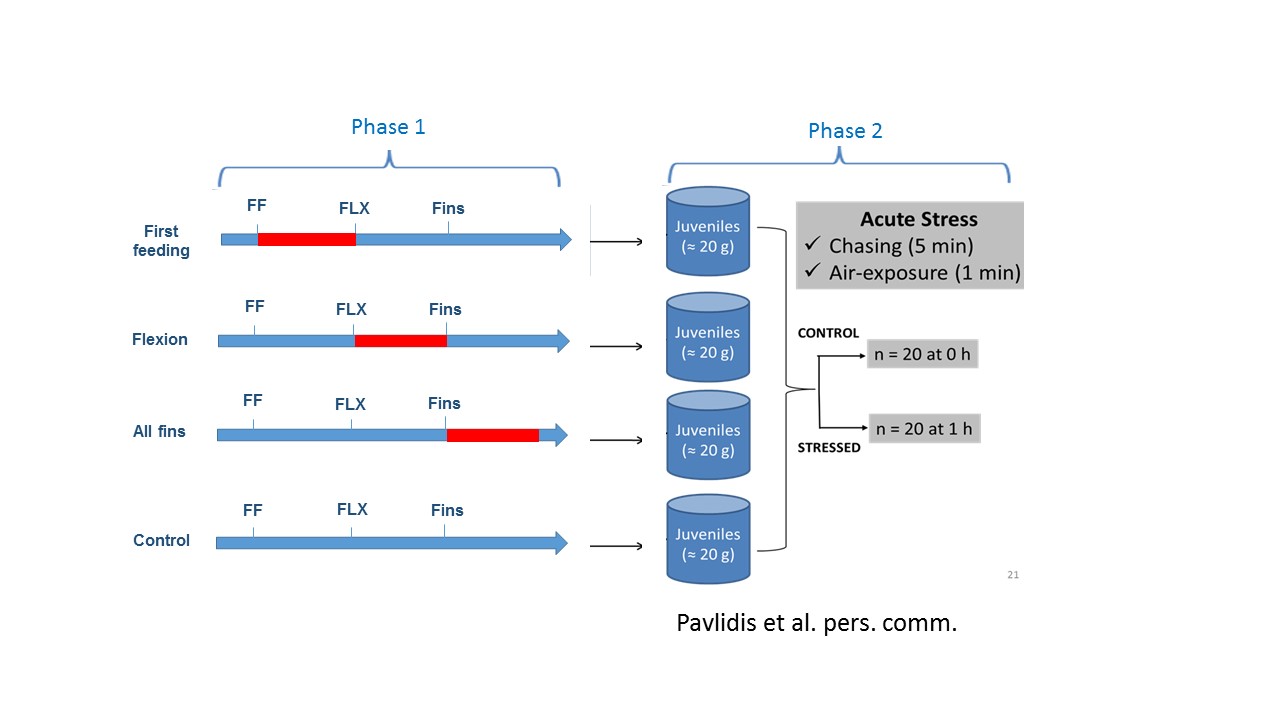

Supplement: Additional file 1: — Experimental set up. Illustration of the experimental set up used in the present study. Red bars indicate the time range where the early-life protocol were applied. FF: first feeding, FLX: first appearance of flexion Fins: first appearance of fins. (JPG 70 kb) [file 12864_2016_2874_MOESM1_ESM.jpg]

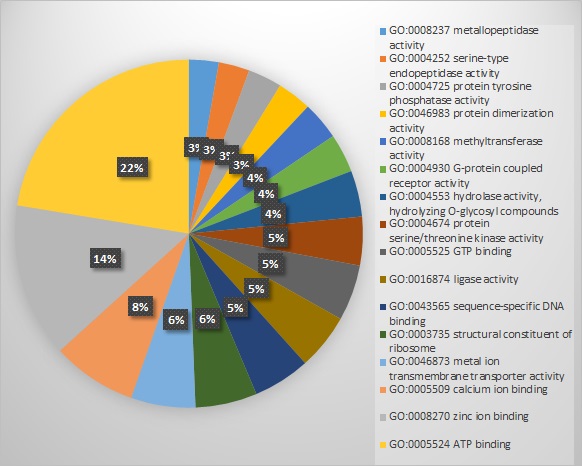

Supplement: Additional file 3: — GO category “Molecular function” of identified transcripts. Summary of GO category “Molecular function” of transcripts significantly expressed in any of the sampling points. (JPEG 81 kb) [file 12864_2016_2874_MOESM3_ESM.jpeg]

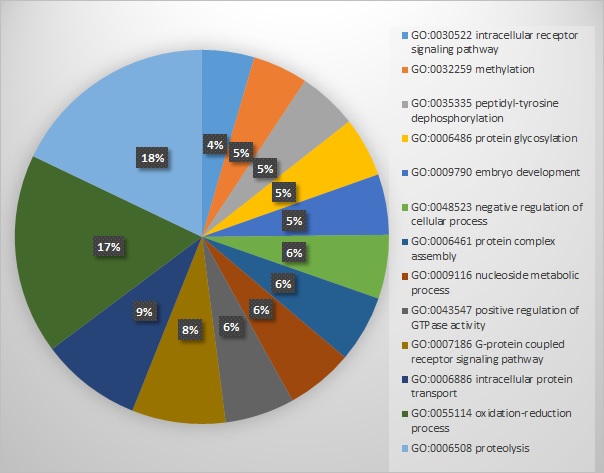

Supplement: Additional file 4: — GO category “Biological process” of identified transcripts. Summary of GO category “Biological process” of transcripts significantly expressed in any of the sampling points. (JPEG 73 kb) [file 12864_2016_2874_MOESM4_ESM.jpeg]
